# The Efficacy and Safety of Technology-Guided Dry Weight Adjustment Among Dialysis Patients: A Meta-analysis of Randomized Controlled Trials

**DOI:** 10.1016/j.xkme.2025.101052

**Published:** 2025-06-17

**Authors:** Wannasit Wathanavasin, Charat Thongprayoon, Paul W. Davis, Wisit Cheungpasitporn

**Affiliations:** 1 Division of Nephrology and Hypertension, Department of Medicine, Mayo Clinic, Rochester, MN; 2 Nephrology Unit, Department of Medicine, Charoenkrung Pracharak Hospital, Bangkok Metropolitan Administration, Bangkok, Thailand

**Keywords:** Bioimpedance, blood volume monitoring, dialysis, lung ultrasound, volume assessment

## Abstract

**Rationale & Objective:**

Recently, various instrumental techniques adjunct to standard clinical evaluation have been used to improve fluid balance and guide dry weight adjustments in dialysis populations. We aimed to explore the efficacy and safety of using technology-guided dry weight adjustment among dialysis patients.

**Study Design:**

Systematic review and meta-analysis of randomized controlled trials (RCTs). The search was conducted in PubMed, Scopus, and Cochrane Central Register of Controlled Trials for relevant RCTs published until April 28, 2024.

**Setting & Participants:**

Adult patients with kidney failure with replacement therapy.

**Exposure:**

Studies with patients receiving technology-guided dry weight adjustments.

**Outcomes:**

Studies were selected if they reported at least 1 outcome of interest (eg, mortality, cardiovascular [CV] events, hospitalization, intradialytic hypotension, hypovolemic events, cardiac arrhythmia, or vascular access problems).

**Analytical Approach:**

Random-effects meta-analyses were conducted, with risk of bias within studies assessed using version 2 of the Cochrane risk-of-bias tool for randomized trials.

**Results:**

A total of 21 RCTs involving 4,239 dialysis patients were analyzed. The meta-analysis revealed that the incorporating technology-guided dry weight adjustment not only was associated with a significant 21% reduction in CV events (relative risk, 0.79; 95% confidence interval [CI], 0.71-0.88) but also resulted in a significant 9% increase in muscle cramps (rate ratio, 1.09; 95% CI, 1.02-1.16). In a subgroup analysis, using bioelectrical impedance analysis was associated with a significant reduction in mortality (relative risk, 0.67; 95% CI, 0.51-0.89). In addition, the intervention led to a significant reduction in pulse wave velocity (mean difference, −2.43 m/s; 95% CI, −4.64 to −0.21).

**Limitations:**

Large number of studies with some concerns or a high risk of bias.

**Conclusions:**

Technology-guided strategies for dry weight adjustment significantly reduce CV events and may lower all-cause mortality in dialysis patients. These benefits are particularly evident with bioelectrical impedance analysis--guided interventions. Nonetheless, clinicians should be aware of a modestly increased risk of muscle cramps.

## Plain-Language Summary

Optimal fluid management in dialysis patients remains challenging in clinical practice. Because clinical evaluation alone has limited sensitivity in detecting subclinical volume overload, several instrumental techniques have been proposed to help more accurately assess dry weight in these patients. This meta-analysis examines the efficacy and safety of using technology-guided dry weight adjustment, including bioelectrical impedance analysis, lung ultrasound, and blood volume monitoring, compared with standard clinical evaluation in dialysis patients. The results showed that the use of these techniques has a positive effect on cardiovascular parameters, particularly pulse wave velocity, and reduces the risk of cardiovascular events in dialysis patients. However, these benefits are accompanied by an increased risk of muscle cramps.

Optimal fluid management in dialysis patients is one of the most essential components of dialysis treatment and remains challenging in clinical practice. Volume overload is common and persists as a significant burden for these patients, being linked to uncontrolled hypertension,^1^ left ventricular hypertrophy,^2^ increased arterial stiffness,^3^ and cardiac dysfunction,^4^ which lead to increased cardiovascular (CV) events and mortality.^5^ In terms of dialysis modalities, despite more continuous fluid removal, peritoneal dialysis (PD) does not result in less volume excess compared with hemodialysis (HD).^6^ As a result, various methods and tools to guide fluid management and alleviate this particular disease burden are being explored among these populations.

Dry weight is traditionally used as a targeted approach for achieving optimal fluid management.^7^ Although volume assessment based on physical examination is the most commonly used method, several studies^8^, ^9^, ^10^ have demonstrated a poor correlation between clinical parameters and fluid overload. Furthermore, clinical assessment alone has limited sensitivity in identifying subclinical volume overload or providing information about fluid distribution.^11^ In this context, several instrumental techniques have been proposed to complement clinical evaluation, helping nephrologists refine clinical assessment and more precisely determine the dry weight of dialysis patients. These techniques include bioelectrical impedance analysis (BIA), lung ultrasound (LUS), and blood volume monitoring (BVM), all of which offer more objective methods for evaluating the complex fluid status in dialysis patients.

Although technology-guided dry weight adjustments are generally considered effective and promising, their impact on improving clinical outcomes, particularly CV outcomes and mortality, remains controversial. Moreover, concerns exist regarding the risk of excessive fluid removal, which can lead to intradialytic hypotension (IDH), hypovolemic events, cardiac arrhythmia, and potential vascular access problems among individuals receiving maintenance dialysis.^12^ Therefore, we conducted this systematic review, incorporating the most recent data from randomized controlled trials (RCTs), to examine the efficacy and safety of using technology-guided dry weight adjustment as an intervention compared with standard clinical evaluation in dialysis patients.

## Methods

This systematic literature review was conducted in compliance with the 2020 Preferred Reporting Items for Systematic Reviews and Meta-Analyses guidelines for reporting interventions. A protocol was preregistered with the PROSPERO database (registration number: CRD 42024576228).

### Searching Strategy

A search of all RCTs comparing adjunct instrumental technique(s) guiding dry weight with standard clinical evaluation in dialysis patients published up to April 28, 2024, was performed by 2 independent reviewers (W.W. and C.T.) on PubMed, Scopus, and Cochrane Central Register of Controlled Trials without language or publication date restrictions. The full search strategy is available in Table S1.

### Inclusion and Exclusion Criteria

We included RCTs with the following criteria: (1) involved dialysis patients aged ≥18 years; (2) compared any intervention of interest (ie, BIA, LUS, or BVM) with standard clinical evaluation; (3) reported on at least one of the following outcomes: all-cause mortality, CV events (a composite of any of the following outcomes: myocardial infarction [fatal or nonfatal], heart failure regardless of left ventricular ejection fraction [LVEF], stroke, and peripheral vascular disease), hospitalization, blood pressure (systolic/diastolic), left ventricular mass index, pulse wave velocity (PWV), IDH, muscle cramp, cardiac arrhythmia, and vascular access problem; (4) had a follow-up duration of at least 3 months; and (5) had a sample size of more than 30 participants. The exclusion criteria comprised the following 3 points: (1) reviews, conference abstracts, and responses to letters; (2) studies involving nonhuman species; and (3) studies involving patients with amputation, implanted metal stents or pacemakers, severe malnutrition, severe heart failure (New York Heart Association Functional Class IV), or chronic liver disease.

### Data Extraction

The assessment of titles and abstracts for each record obtained, as well as the examination of full-text reports, was carried out independently by 2 reviewers (W.W. and C.T.). Whenever a discrepancy arose between the 2 reviewers, resolution was achieved through discussion involving the third author (W.C.). In case of duplicated studies of the same patient population, the study reporting the higher number of participants was selected as the main data source. For studies that reported outcomes at multiple time points, the time point with the longest follow-up was selected. The following data were extracted from each report, including the first author, year of publication, research country, participant numbers, patients’ characteristics, type of intervention, outcomes of interest, duration of follow-up, and risk of bias score.

### Assessment of Quality and Risk of Bias

The risk of bias in each included study was also independently assessed by 2 reviewers (W.W. and C.T.) using the Cochrane risk-of-bias tool for randomized trials (RoB 2)^13^ as recommended by Cochrane, 2022. Any discrepancies were resolved by the third author (W.C.).

### Statistical Analysis

All statistical analyses were performed using Stata statistical package version 16 (StataCorp). Statistical significance was determined with a 2-tailed *P* value of less than 0.05. The meta-analysis was performed by using random-effects model to estimate the pooled-effect estimates with their 95% confidence intervals (95% CIs). For dichotomous outcomes, results were summarized by the pooled relative risk (RR) with 95% CI. For outcomes reported as counts, indicating events that could occur multiple times in a single patient, the results were reported as pooled rate ratios with 95% CI. For outcomes reported on a continuous scale, results were reported as weighted mean differences (WMD) with 95% CI.

Heterogeneity was assessed using the *I*^2^ statistic and Q-test *P* value. The *I*^2^ index ranges between 0% and 100%. *I*^2^ values of 25%, 50%, and 75% have been suggested to indicate low, moderate, and high heterogeneity, respectively. To explore sources of heterogeneity, we performed subgroup meta-analyses according to continents, dialysis modality, dialysis vintage, type of intervention, follow-up duration, and risk of bias of the study. To graphically represent this heterogeneity among the included studies, a forest plot was employed. Publication bias was assessed formally using Funnel plots and the Egger test.

### Grading the Strength of Evidence

W.W. and C.T. individually graded the strength of evidence for the outcomes of interest by considering the risk of bias of each study, inconsistency of the results, indirectness of evidence, imprecision, and reporting bias following the Grading of Recommended Assessment, Development, and Evaluation approach.^14^

## Results

### Search Results

**Figure 1:**
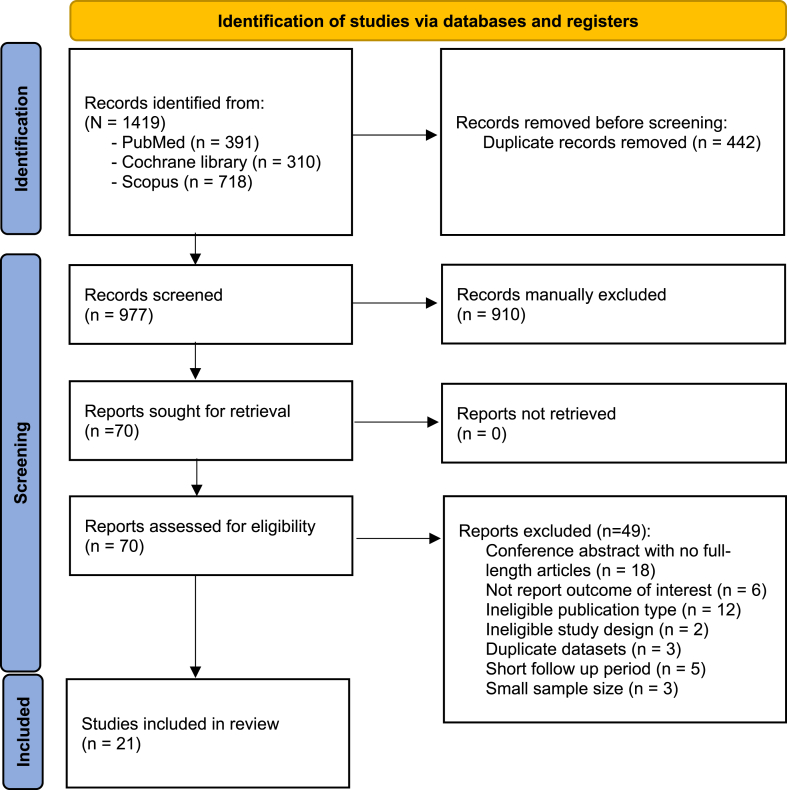
Preferred Reporting Items for Systematic Reviews and Meta-Analyses 2020 flow diagram.

A total of 1,419 potentially relevant articles were initially identified through the database search. After removing 442 duplicated articles, the titles and abstracts were screened based on the inclusion and exclusion criteria, resulting in the identification of 70 full-text publications that underwent subsequent evaluation. Following full-text screening, 49 articles were excluded for reasons detailed in Fig 1. Finally, 21 RCTs were included in the systematic review and meta-analysis (Fig 1).

### Characteristics of Included Studies

Detailed characteristics and key features of individual studies are shown in Table 1.^15^, ^16^, ^17^, ^18^, ^19^, ^20^, ^21^, ^22^, ^23^, ^24^, ^25^, ^26^, ^27^, ^28^, ^29^, ^30^, ^31^, ^32^, ^33^, ^34^, ^35^ There were 21 RCTs, with a total of 4,239 dialysis patients (3,094 maintenance HD patients and 1,145 PD patients). Among the included patients, the mean age was 58.6 years, 56.1% were men, and 28.5% had diabetes mellitus whereas 75.7% had hypertension as comorbid conditions. The mean dialysis vintage was 46.1 months. The following 3 instrumental techniques were used for dry weight adjustment interventions: (1) BIA, (2) LUS, and (3) BVM.

**Table 1:** Characteristics of the Studies Included in the Systematic Review

No.	Author	Year of Publication	Country	No. of Patients	Mode of KRT	RKF (mL/d)	Mean Age (y)	Men (%)	DM (%)	HT (%)	Dialysis Vintage (mo)	Intervention	Clinical Outcomes of Interest	CV Parameters of Interest	Adverse Events of Interest	FU Time (mo)	Risk of Bias
1	Reddan et al^15^	2005	United States/ Canada	443	HD	NA	59.2	51	44.7	88.2	NA	BVM	Mortality, hospitalization	SBP, DBP	IDH, muscle cramp	6	Low
2	Luo et al^16^	2011	China	160	PD	778.6	60	46.3	27.5	NA	34.2	BIA	NA	SBP, DBP	NA	3	High
3	Onofriescu et al^17^	2012	Romania	135	HD	NA	52.4	51.1	10.3	51.5	51	BIA	NA	SBP, DBP, PWV	NA	12	High
4	Hur et al^18^	2013	Turkey	156	HD	NA	51.6	55.8	NA	NA	61.85	BIA	Mortality, CV events, hospitalization	SBP, DBP, PWV, LVMI	IDH	12	Some concerns
5	Onofriescu et al^19^	2014	Romania	131	HD	NA	53.1	53	9.5	69.2	105.4	BIA	Mortality	PWV, SBP	IDH	30	Low
6	Tan et al^20^	2016	United Kingdom/China	308	PD	NA	55.9	51.3	NA	NA	31.8	BIA	Mortality	SBP, DBP	NA	12	Low
7	Huan-Sheng et al^21^	2016	Taiwan	298	HD	NA	62.4	48.7	NA	NA	NA	BIA	Mortality, CV events, hospitalization	NA	IDH, muscle cramp, cardiac arrhythmia	12	Some concerns
8	Siripol et al^31^	2017	Romania	250	HD	NA	59.2	46.4	19.2	76	43.4	BIA + LUS	Mortality, CV events, hospitalization	PWV	IDH, muscle cramp, VA problem	21.3	Some concerns
9	Oh et al^32^	2018	Korea	137	PD	NA	51.7	54	33.6	NA	25.2	BIA	CV events	SBP, DBP, PWV, LVMI	NA	12	Low
10	Yoon et al^22^	2019	Korea	201	PD	>500	54.6	55.7	51.7	83.6	NA	BIA	Mortality, CV events	SBP, DBP, LVMI	NA	3	High
11	Patel et al^33^	2019	India	50	HD	NA	56	70	NA	NA	29.2	BIA	NA	SBP, DBP	IDH, muscle cramp	6	Some concerns
12	Liu et al^23^	2020	China	445	HD	>800	54.8	53.9	23.7	NA	49.2	BIA	Mortality, CV events	NA	NA	13.6	High
13	Tian et al^24^	2020	China	240	PD	250	49	51	21	NA	32	BIA	Mortality, CV events	SBP, DBP	IDH	36	Some concerns
14	Sommerer et al^25^	2021	Germany	132	HD	799	70	58.3	26.5	24.2	59	BIA	NA	SBP, DBP	IDH, muscle cramp, cardiac arrhythmia, VA problem	3	Some concerns
15	Loutradis et al^34^	2021	Greece/ Slovania	71	HD	444	62.4	66.2	26.8	100	33.6	LUS	NA	SBP, DBP	IDH	12	Low
16	Zoccali et al^26^	2021	European	363	HD	NA	70	70.2	40.5	75.5	55	LUS	Mortality, CV events	LVMI	IDH, cardiac arrhythmia	17.88	Low
17	Brimble et al^27^	2022	Canada	65	PD	1,005.7	61.6	52	39	92	20.4	BIA	Mortality, CV events, hospitalization	SBP, DBP, LVMI	NA	12	High
18	Loutradis et al^35^	2022	Greece/ Slovania	71	HD	444	62.4	66.2	26.8	100	33.6	LUS	NA	LVMI	IDH	12	Some concerns
19	Costa et al^28^	2022	Brazil	34	PD	1,137.1	58	57.4	38.3	100	106.8	BIA	NA	PWV, LVMI	NA	9	High
20	Stigger et al^29^	2023	Brazil	110	HD	NA	57.4	58	34.5	61.8	30.7	BIA	Mortality, hospitalization	SBP, DBP	Muscle cramp	24	Some concerns
21	Davies et al^30^	2023	United Kingdom	439	HD	>500	61.6	70.3	12.9	NA	NA	BIA	Mortality	NA	NA	24	Low

Abbreviations: BIA, bioimpedance analysis; BVM, blood volume monitoring; CV events, cardiovascular events; DBP, diastolic blood pressure; DM, diabetes mellitus; FU, follow-up; HD, hemodialysis; HT, hypertension; IDH, intradialytic hypotension; KRT, kidney replacement therapy; LUS, lung ultrasound; LVMI, left ventricular mass index; NA, not applicable; PD, peritoneal dialysis; PWV, pulse wave velocity; RKF, residual kidney function; SBP, systolic blood pressure; VA, vascular access.

### Risk of Bias and Grading of Recommended Assessment, Development, and Evaluation Assessment

**Figure 2:**
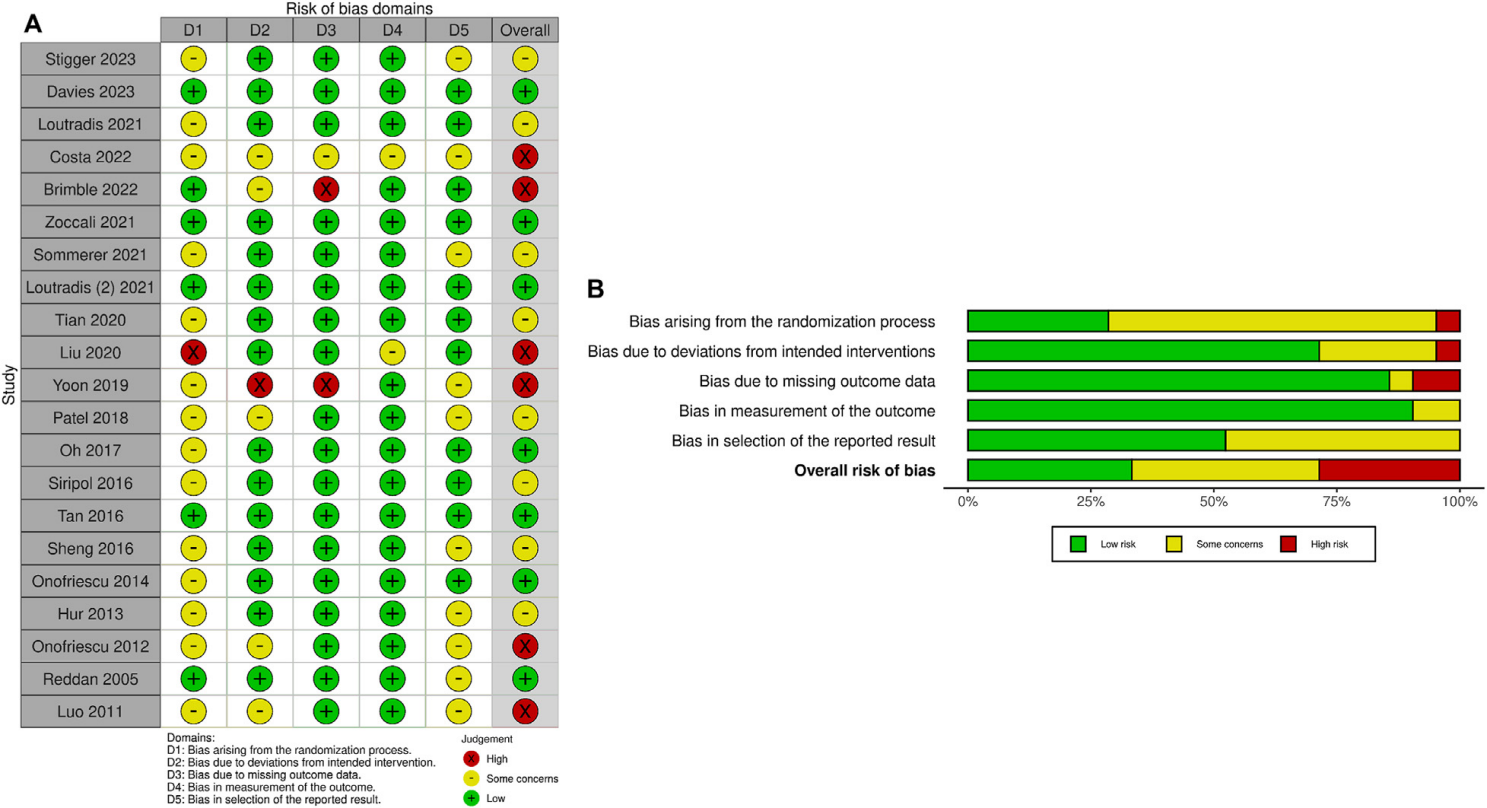
Risk of bias assessment of included randomized controlled trials. (A) A traffic light plot and (B) a weighted summary plot of the overall type of bias.

According to RoB2,^13^ 7 studies had a low risk of bias, 7 had a high risk of bias, and 8 had some concerns of bias. Figure 2 shows details on the rating of each RoB2 domain. The overall assessment showed a moderate bias (Fig 2A and B). Grading of Recommended Assessment, Development, and Evaluation assessments for the efficacy and safety outcomes ranged from very low to moderate (Table 2). Further details about Grading of Recommended Assessment, Development, and Evaluation are presented in Table S2.

**Table 2:** Summary of Findings for Efficacy and Safety Outcomes

Outcomes	No. of Studies	No. of Patients			Effect Estimate (95% CI)	*I*^2^	GRADE Evidence
		Total	Intervention	Control			
**Efficacy**							
Clinical outcomes							
Mortality	13	3,448	1,741	1,707	RR 0.82 (0.63-1.07)	35.7%	⨁⨁⨁◯ Moderate
CV events	9	2,155	1,090	1,065	RR 0.79 (0.71-0.88)	0%	⨁⨁◯◯ Low
Hospitalization	5	1,166	588	578	RR 1.03 (0.92-1.16)	0%	⨁◯◯◯ Very low
Cardiovascular parameters							
SBP (mm Hg)	15	1,829	908	921	WMD −1.97 (−4.69, 0.75)	73.14%	⨁◯◯◯ Very low
DBP (mm Hg)	14	1,698	846	852	WMD −0.63 (−2.10, 0.85)	62.98%	⨁◯◯◯ Very low
LVMI (g/m^2^) indexed for BSA	6	608	306	302	WMD −3.33 (−8.36, 1.71)	0%	⨁⨁◯◯ Low
LVMI (g/m^2^) indexed for height	2	410	207	203	WMD −0.83 (−4.64, 2.98)	0%	⨁⨁◯◯ Low
LVEF (%)	6	815	411	404	WMD +1.13 (−0.08%, 2.34%)	0%	⨁⨁◯◯ Low
PWV (m/s)	6	847	421	426	WMD −2.43 (−4.64, −0.21)	98.84%	⨁⨁◯◯ Low
**Safety**							
Adverse events							
IDH	5	1,086	542	544	Rate ratio 0.93 (0.83-1.04)	74.83%	⨁◯◯◯ Very low
Muscle cramp	5	1,166	588	578	Rate ratio 1.09 (1.02-1.16)	0%	⨁⨁◯◯ Low
Cardiac arrhythmia	3	786	394	392	Rate ratio 0.95 (0.64-1.39)	0%	⨁⨁◯◯ Low
VA problems	4	1,036	517	519	Rate ratio 1.13 (0.89-1.43)	0%	⨁⨁◯◯ Low

Abbreviations: BSA, body surface area; CI, confidence interval; CV events; cardiovascular events; DBP, diastolic blood pressure; GRADE, Grading of Recommended Assessment, Development, and Evaluation approach; IDH, intradialytic hypotension; LUS, lung ultrasound; LVMI, left ventricular mass index; PD, peritoneal dialysis; PWV, pulse wave velocity; RR, relative risk; SBP, systolic blood pressure; VA, vascular access; WMD, weighted mean difference.

### Efficacy of Technology-Guided Dry Weight Adjustment in Clinical Outcomes

#### All-Cause Mortality

**Figure 3:**
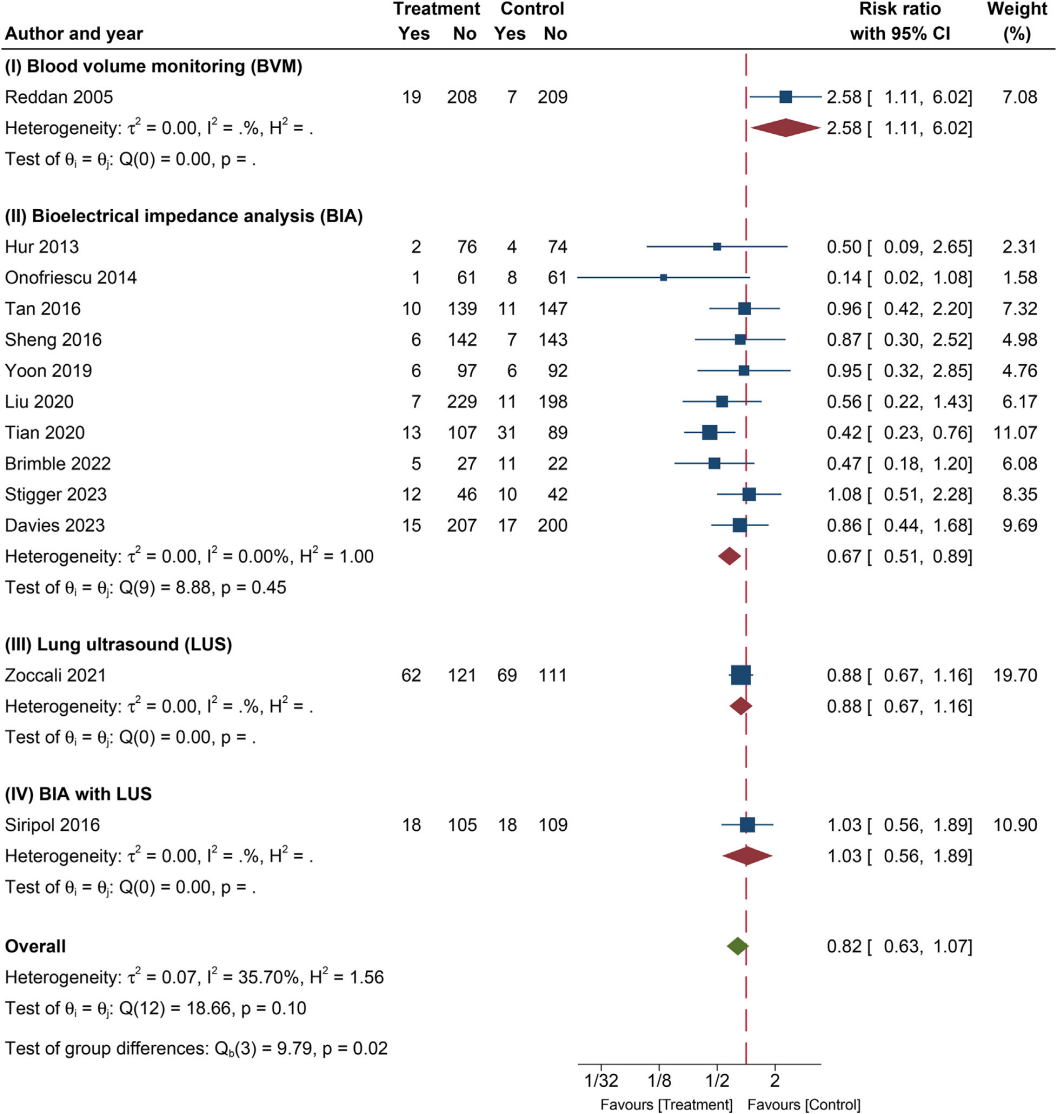
A forest plot of the effect on all-cause mortality in different types of intervention. Abbreviation: CI, confidence interval.

The use of technology-guided dry weight adjustment demonstrated a trend toward reducing mortality (pooled RR, 0.82; 95% CI, 0.63-1.07; *P* = 0.14; *I*^2^, 35.7%; Fig 3 and Table 2) compared with standard clinical evaluation, although pooled estimates did not reach statistical significance.^15^^,^^18^, ^19^, ^20^, ^21^, ^22^, ^23^, ^24^^,^^26^^,^^27^^,^^29^, ^30^, ^31^

#### CV Events

**Figure 4:**
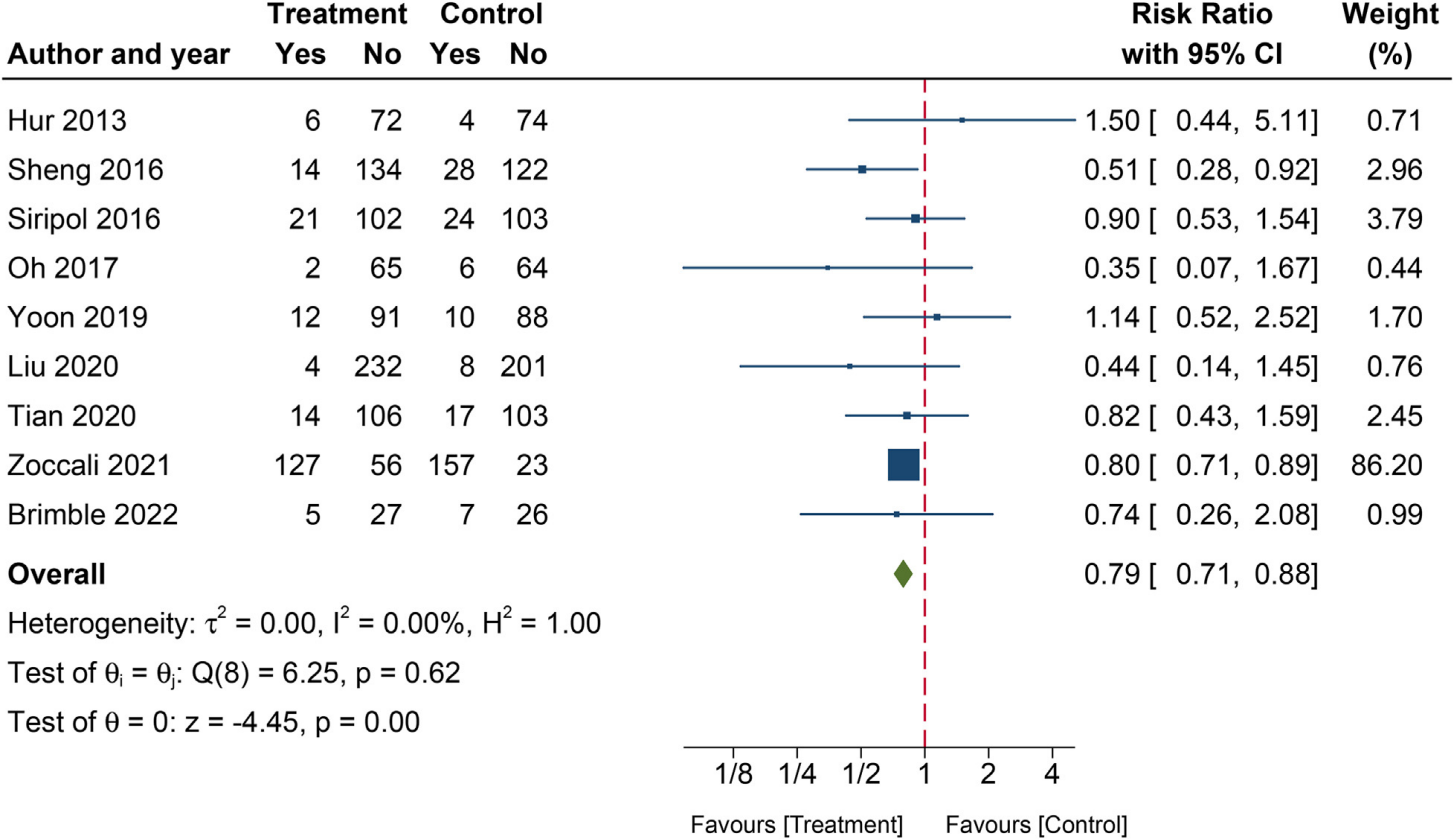
A forest plot of the effect of technology-guided dry weight adjustment on cardiovascular events. Abbreviation: CI, confidence interval.

The results demonstrated that the use of technology-guided dry weight adjustment significantly lowered CV events (pooled RR, 0.79; 95% CI, 0.71-0.88; *P* < 0.001; *I*^2^, 0%; Fig 4 and Table 2) compared with standard clinical evaluation.^18^^,^^21^, ^22^, ^23^, ^24^^,^^26^^,^^27^^,^^31^^,^^32^

#### Hospitalization

There was no significant difference in hospitalization rate (pooled RR, 1.03; 95% CI, 0.71-0.88; *P* = 0.59; *I*^2^, 0%; Table 2) when comparing the use of technology-guided dry weight adjustment with standard clinical evaluation.^15^^,^^21^^,^^27^^,^^29^^,^^31^

### Efficacy of Technology-Guided Dry Weight Adjustment in CV Parameter Outcomes

#### Systolic Blood Pressure

The results showed that the use of technology-guided dry weight adjustment did not significantly reduce systolic blood pressure (SBP) (WMD, −1.97 mm Hg; 95% CI, −4.69 to 0.75; *P* = 0.16; *I*^2^, 73.14%; Table 2) compared with standard clinical evaluation.^16^, ^17^, ^18^, ^19^, ^20^^,^^22^^,^^24^^,^^25^^,^^27^^,^^29^^,^^32^, ^33^, ^34^

#### Diastolic Blood Pressure

The use of technology-guided dry weight adjustment did not significantly reduce diastolic blood pressure (DBP) (WMD, −0.63 mm Hg; 95% CI, −2.10 to 0.85; *P* = 0.41; *I*^2^, 62.98%; Table 2) compared with standard clinical evaluation.^16^, ^17^, ^18^^,^^20^^,^^22^^,^^24^^,^^25^^,^^27^^,^^29^^,^^32^, ^33^, ^34^

#### Left Ventricular Mass Index

The results showed that the use of technology-guided dry weight adjustment did not significantly reduce the indexation of left ventricular mass to body surface area and height (WMD, −3.33 g/m^2^; 95% CI, −8.36 to 1.71; *P* = 0.20; *I*^2^, 0% and WMD, −0.83 g/m^2^, 95% CI, −4.64 to 2.98; *P* = 0.67; *I*^2^, 0%, respectively; Table 2) compared with standard clinical evaluation.^18^^,^^22^^,^^26^, ^27^, ^28^^,^^32^^,^^35^

#### LVEF

Meta-analysis showed no significant difference in LVEF improvement by the use of technology-guided dry weight adjustment (WMD, +1.13%; 95% CI, −0.08 to 2.34; *P* = 0.07; *I*^2^, 0%; Table 2) compared with standard clinical evaluation.^22^^,^^26^, ^27^, ^28^^,^^32^^,^^35^

#### PWV

The use of technology-guided dry weight adjustment significantly reduced PWV (WMD, −2.43 m/s; 95% CI, −4.64 to −0.21; *P* < 0.001; *I*^2^, 98.84%; Table 2) compared with standard clinical evaluation.^17^, ^18^, ^19^^,^^28^^,^^31^^,^^32^

### Safety of Technology-Guided Dry Weight Adjustment

#### IDH

The results showed that the use of technology-guided dry weight adjustment did not significantly increase the risk of IDH (pooled rate ratio, 0.93; 95% CI, 0.83-1.04; *P* = 0.22; *I*^2^, 78.3%; Table 2) compared with standard clinical evaluation.^21^^,^^25^^,^^26^^,^^31^^,^^33^

#### Muscle Cramp

The use of technology-guided dry weight adjustment significantly increased the risk of muscle cramp (pooled rate ratio, 1.09; 95% CI, 1.02-1.16; *P* = 0.01; *I*^2^, 0%; Table 2) compared with standard clinical evaluation.^21^^,^^25^^,^^29^^,^^31^^,^^33^

#### Cardiac Arrhythmia

The results showed that the use of technology-guided dry weight adjustment did not significantly increase the risk of cardiac arrhythmia (pooled rate ratio, 0.95; 95% CI, 0.64-1.39; *P* = 0.64; *I*^2^, 0%; Table 2) compared with standard clinical evaluation.^21^^,^^25^^,^^26^

#### Vascular Access Problems

The use of technology-guided dry weight adjustment did not significantly increase the risk of vascular access problems (pooled rate ratio, 1.13; 95% CI, 0.89-1.43; *P* = 0.32; *I*^2^, 0%; Table 2) compared with standard clinical evaluation.^21^^,^^25^^,^^26^^,^^31^

### Investigation of Heterogeneity

We observed substantial heterogeneity in the pooled-effect estimates among studies for 5 outcomes. These included 2 studies examining clinical outcomes, including mortality and IDH (*I*^2^ = 35.7% and 74.8%, respectively), and 3 studies focusing on CV parameter outcomes, including SBP, DBP, and PWV (*I*^2^ = 73.1%, 63%, and 98.8%, respectively). The remaining outcomes showed no evidence of heterogeneity across the studies.

Table 3 and Tables S3-S7 detail the results of subgroup analyses. In brief, technology-guided dry weight adjustment significantly reduced all-cause mortality in subgroups of PD patients and the Asian population compared with standard clinical evaluation. In the subgroup analysis of dry weight assessment tools, BIA-guided dry weight adjustment, in particular, was significantly linked to reducing all-cause mortality (RR, 0.67; 95% CI, 0.51-0.89; *P* for interaction = 0.02). In contrast, BVM was associated with an increased risk of all-cause mortality (RR, 2.58; 95% CI, 1.11-6.02) compared with standard clinical evaluation from a single study (Table 3).

**Table 3:** Subgroup Analysis Examining the Effect of Technology-Guided Dry Weight Adjustment on Mortality Outcomes

Subgroup Analyses	No. of Studies	No. of Patients	Relative Risks (95% CI)	*P* Value	Assessment of Heterogeneity	
					*I*^2^ Index	*P* Value
Dialysis modalities						
PD	4	813	0.59 (0.38-0.93)	0.02	15.91%	0.31
HD	9	2,635	0.93 (0.70-1.25)	0.64	27.22%	0.20
Race						
Asian	5	1,340	0.56 (0.37-0.84)	<0.01	0%	0.65
Non-Asian	7	1,801	0.94 (0.65-1.34)	0.73	47.27%	0.08
Mixed	1	307	0.96 (0.42-2.20)	0.93	0%	1.00
Tools of dry weight assessment (*P* = 0.02^a^)						
BVM	1	443	2.58 (1.11-6.02)	0.03	0%	1.00
BIA	10	2,392	0.67 (0.51-0.89)	0.01	0%	0.45
LUS	1	363	0.88 (0.67-1.16)	0.38	0%	1.00
BIA + LUS	1	250	1.03 (0.56-1.89)	0.92	0%	1.00
Study follow-up time						
≤1 y	5	1,269	0.95 (0.50-1.81)	0.88	50.42%	0.09
>1 y	8	2,179	0.77 (0.59-1.02)	0.07	28.41%	0.20
Dialysis vintage						
Incident	2	445	0.75 (0.43-1.28)	0.29	0%	0.47
Prevalent	11	3,003	0.83 (0.61-1.14)	0.26	44.23%	0.06
Risk of bias						
High	3	711	0.61 (0.34-1.07)	0.08	0%	0.62
Low	5	1,683	0.99 (0.61-1.61)	0.97	55.93%	0.06
Some concerns	5	1,054	0.75 (0.48-1.16)	0.19	32.27%	0.21

Abbreviations: BIA, bioimpedance analysis; CI, confidence interval; BVM, blood volume monitoring; HD, hemodialysis; LUS, lung ultrasound; PD, peritoneal dialysis.

a Statistical significance.

The effect of technology-guided dry weight adjustment on the rate of IDH was consistent across different races and follow-up durations. However, a decreased risk of IDH was observed in the subgroup of studies with a low risk of bias and among patients who received LUS-based fluid management. The use of technology-guided dry weight adjustment significantly decreased both SBP and DBP in the Asian compared with non-Asian population. In addition, a reduction in SBP was observed in studies with a low risk of bias and a follow-up duration of 1 year or less. In contrast, an increase in DBP was observed in studies with a follow-up duration of more than 1 year. Last, the effect of technology-guided dry weight adjustment on reducing PWV was consistent across different races, study risk of bias, and follow-up durations. However, a significant increase in PWV was observed in patients who received BIA combined with LUS-based fluid management.

### Assessment of Publication Bias

**Figure 5:**
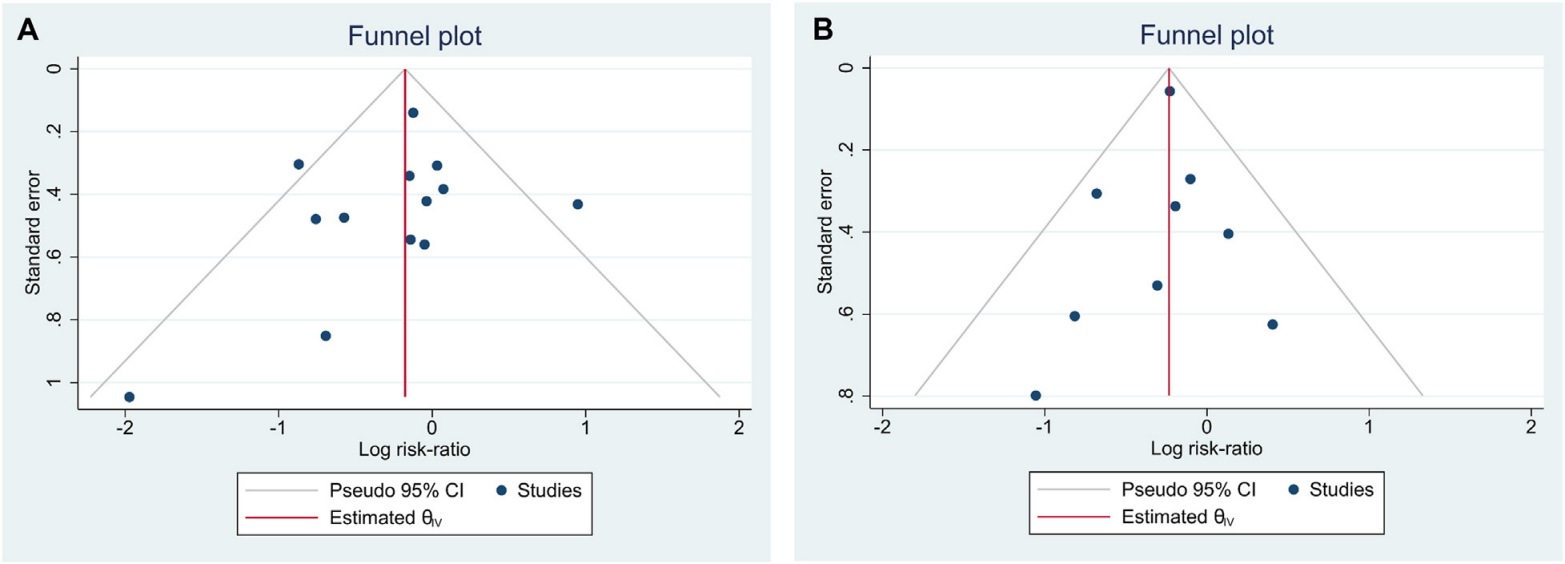
Funnel plots of individual studies displaying the standard error based on the log risk ratio for (A) all-cause mortality and (B) cardiovascular event outcomes; *P* = 0.31 and 0.69, respectively, based on the Egger test. Abbreviation: CI, confidence interval.

Publication bias was evaluated using the Egger regression model. The Funnel plot for the outcomes, including all-cause mortality, CV events, SBP, and DBP in the studies included in the meta-analysis, was symmetrical (Fig 5A and B and Figs S1 and S2). The results of the Egger test did not show a significant publication bias for any of the outcomes.

## Discussion

This systematic review and meta-analysis aimed to provide comprehensive and updated evidence regarding the efficacy and safety of instrumental techniques, in addition to standard clinical evaluation, for dry weight adjustment in long-term dialysis patients, including their impact on clinical outcomes and CV parameters. We mainly focused on available RCTs and provided a summary of the evidence pooled from 21 studies, including a total of 4,239 dialysis patients. Our analysis demonstrated that implementing technology-guided dry weight adjustment has a beneficial impact on CV parameters, particularly in reducing PWV, and decreases the risk of CV events in dialysis patients. Moreover, the application of BIA-guided fluid control was significantly associated with a reduced risk of all-cause mortality. However, these benefits are accompanied by an increased risk of hypovolemic events, in particular, muscle cramps.

Persistent volume overload has long been recognized as a significant and independent risk factor for all-cause mortality and CV events in dialysis patients.^4^ The decades-old strategy of relying solely on clinical evaluation has not been effective in reducing the notoriously high CV events and mortality in these patients. As a result, over the past 2 decades, several emerging techniques for managing fluid status have advanced significantly beyond clinical assessment, using more objective approaches. Remarkably, our analysis revealed a significant reduction in overall CV events with the implementation of instrument-guided dry weight adjustment across all subgroups (Table 2, Table S3, and Fig 4). This finding may be attributed to improvements in some CV parameters, such as PWV and LVEF (Table 2). According to Tripepi et al,^36^ arterial stiffness, as measured based on PWV, is prevalent and regarded as a strong indicator of arterial damage, predicting a poor CV prognosis among dialysis patients. In addition, Miseljic et al^37^ proved that significantly higher values of PWV were found in overhydrated participants, which indicates volume overload. Therefore, the observed reduction in PWV in the group using adjunct technologies for dry weight adjustment, as demonstrated in our analysis and supported by the meta-analysis published in 2023,^38^ could signify an improvement in volume overload, potentially leading to a decrease in CV events.

Recently, LVEF has been the most frequently used echocardiographic parameter to define left ventricular systolic function and serves as a negative predictor of major CV events in dialysis patients. According to a study by Tseng et al,^39^ a poor LVEF of 45% or lower was significantly associated with a higher incidence of major CV events in both PD and HD patients, with a hazard ratio of 2.20 (95% CI, 1.47-3.30; *P* < 0.001). In addition, Hong et al^40^ revealed that LVEF in PD patients with time-averaged overhydration, monitored using bioimpedance spectroscopy, was significantly lower than that in those with time-averaged normohydration at 12 months (β = −0.190; *P* = 0.010). In another study, Chan et al^41^ demonstrated that nocturnal HD, which theoretically provides better volume control than conventional HD, leads to a sustained and significant increase in LVEF of approximately 13% (*P* = 0.01). This implies that adequate fluid management plays an important role in reducing CV risk in dialysis patients. Consistent with our findings, the use of technology-guided dry weight adjustment for fluid control tended to improve LVEF (WMD, +1.13%; *P* = 0.07) compared with standard clinical evaluation. However, the pooled estimates did not achieve statistical significance, possibly due to insufficient statistical power.

With respect to the impact of technology-guided dry weight adjustment on all-cause mortality, the present meta-analysis found that this approach did not significantly lower all-cause mortality compared with standard clinical assessment, with moderate heterogeneity (Table 2). To address this heterogeneity, we conducted a subgroup analysis that highlighted that these tools were beneficial in reducing mortality specifically among individuals receiving BIA-based fluid management (*P* for interaction = 0.02) (Fig 3). Among various technology-guided methods, BIA is the most commonly used tool for assisting volume management owing to its appealing features of being simple, inexpensive, and noninvasive.^42^^,^^43^ Compared with other methods, BIA appears to be a more objective approach than LUS and BVM, which are operator dependent, lack standardization, and require specific training.^43^^,^^44^ In addition, almost all forms of BIA included in the present meta-analysis were whole body and multifrequency, which appear to be more accurate than other forms, because they incorporate underlying physical principles into their equations.^45^ Corresponding to our findings of a lower mortality risk with BIA-guided management, 2 previous meta-analyses^38^^,^^46^ provide similar support. These findings suggest that among the various mortality risk factors for dialysis patients,^47^^,^^48^ achieving appropriate fluid control to optimal dry weight, meticulously guided by suitable instruments, is crucial and clinically significant. Despite that, not all instrument-guided fluid management methods are identical, as mentioned above and illustrated in Fig 3. Although prior meta-analyses demonstrate comparable mortality findings, one of the studies^46^ did not identify the source of heterogeneity through subgroup analysis. It is worth noting that factors such as dialysis modalities and race might influence the pooled-effect estimate of various clinical outcomes. Another study by Yang et al^38^ solely evaluated mortality and did not explore the effects on CV outcomes or hospitalization. Including these outcomes would offer further insights into the etiological factors contributing to increased mortality.

In terms of safety, the incidence of serious adverse events, including IDH, cardiac arrhythmia, and vascular access problems, was similar between the tool-guided dry weight adjustment group and the standard clinical evaluation group. However, some concerns related to hypovolemic events, particularly muscle cramps, were noted to be more frequent in the intervention group (Table 2). This implies that beyond reaching the appropriate dry weight, gradual fluid removal during HD should be considered to prevent hypovolemic events, particularly in diabetic and elderly dialysis patients with a poor plasma refill rate.^49^ Of note, the concept of maintaining euvolemia, avoiding both overhydration as well as underhydration, remains a fundamental objective of dialysis therapy.^12^

Our meta-analysis has several strengths. We provide the most up-to-date and largest evidence on efficacy, encompassing clinical outcomes and essential CV parameters, as well as safety outcomes for current techniques used in assessing fluid status in dialysis patients. To ensure result accuracy, we rigorously selected only RCTs and used exclusively valid data without imputation. In contrast to prior meta-analyses^38^^,^^46^ focusing solely on efficacy, our study equally emphasizes safety outcomes, recognizing their critical importance in volume management for dialysis patients. Our findings reveal varying effects on outcomes based on dialysis modality and intervention type, potentially guiding nephrologists in tailoring management strategies to individual patients.

It is important to acknowledge some potential limitations of this meta-analysis. Regarding the inclusion criteria, all studies included were RCTs that used instrument-assisted dry weight adjustment interventions, which could not be completely blinded in some studies. Consequently, subjective factors may contribute to a certain degree of bias in quality assessment. Moreover, most of the included studies had RoB issues and were rated as having some concerns or high risk. Consequently, the quality of the pooled evidence is limited by the quality of data used for syntheses. Although our analysis provides valuable insights, several key questions remain unanswered. Notably, only 1 RCT involving BVM was included in our analysis. Although our results suggest limited efficacy of BVM-guided dry weight adjustment, this finding should be interpreted with caution. The paucity of RCTs evaluating BVM limits the generalizability of this conclusion. Future studies—including well-designed nonrandomized trials and real-world evidence—may help further elucidate the role of BVM in dialysis care. In addition, large-scale, multicenter RCTs with extended follow-up periods are needed to definitively establish the long-term impact of technology-guided dry weight adjustment on important patient outcomes and to provide more robust clinical evidence.

## Conclusions

The present meta-analysis demonstrated the benefits of instrumental techniques, in addition to standard clinical evaluation, for dry weight adjustment on CV parameters, particularly PWV, and a reduction in the risk of CV events among dialysis patients. However, these benefits are accompanied by an increased risk of muscle cramps.
