# Supplementary material for: The Efficacy and Safety of Technology-Guided Dry Weight Adjustment Among Dialysis Patients: A Meta-analysis of Randomized Controlled Trials
Source: Kidney Med. 2025 Jun 17;7(8):101052. doi: 10.1016/j.xkme.2025.101052 (PMC12309956; doi:10.1016/j.xkme.2025.101052)
Supplement: Supplementary File (PDF) — Figures S1 and S2; Tables S1-S7. [file mmc1.pdf]

Figure S1. Funnel plot of individual studies displaying the standard error by the log risk ratio for systolic blood pressure, P = 0.79 by the Egger test

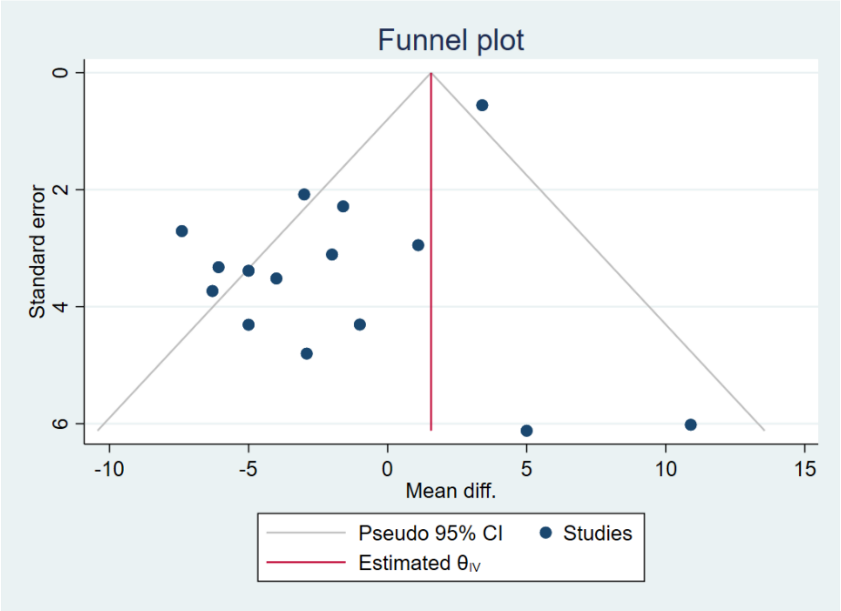

Figure S2. Funnel plot of individual studies displaying the standard error by the log risk ratio for diastolic blood pressure, P = 0.33 by the Egger test

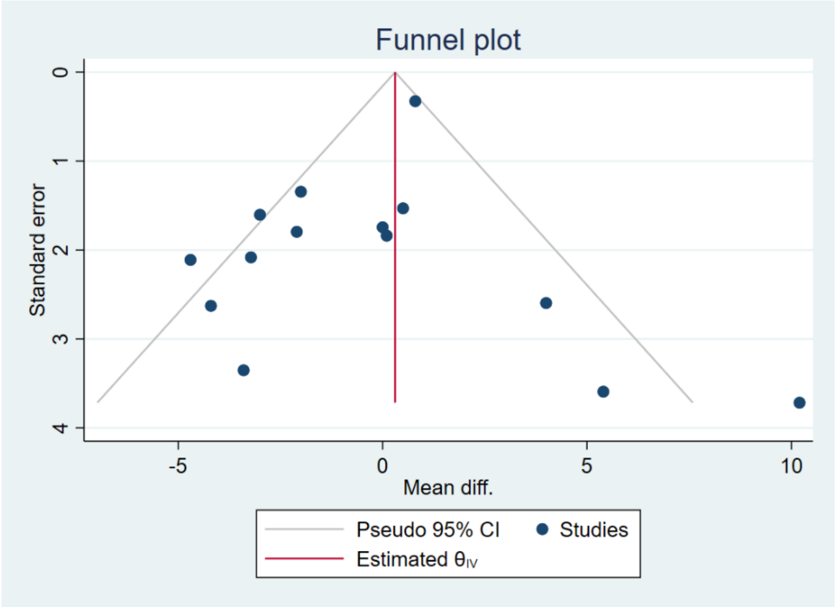

Table S1. Keywords for article searches

Keywords	No. of articles
Pubmed	391
Population of interest<br>"dialysis"[Text Word] OR "hemodialysis" [Text Word] OR "haemodialysis" [Text Word] OR "peritoneal dialysis"[Text Word] OR "renal replacement therapy"[Text Word] OR "end stage renal failure"[Text Word] OR "esrf"[Text Word] OR "end stage renal disease"[Text Word] OR "esrd" [Text Word] OR "kidney failure"[Text Word] OR "renal insufficiency"[Text Word] OR "kidney insufficiency"[Text Word] OR "Renal Dialysis"[MeSH Terms] OR "renal insufficiency, chronic"[MeSH Terms]<br>**AND**<br>*Intervention of interest*<br>"volume assessment" [Text Word] OR "volume management"[Text Word] OR "volume control"[Text Word] OR "volume overload"[Text Word] OR "fluid overload"[Text Word] OR "lung congestion"[Text Word] OR "hypervolemia"[Text Word] OR "overhydration"[Text Word] OR "fluid status"[Text Word] OR "weight adjustments"[Text Word] OR "hydration status"[Text Word] OR "target weight"[Text Word] OR "dry weight"[Text Word] OR "goal weight"[Text Word] OR "ideal weight"[Text Word] AND "bioimpedance"[Text Word] OR "bio-impedance"[Text Word] OR "bioimpedance analysis"[Text Word] OR "bio-impedance analysis"[Text Word] OR "bia"[Text Word] OR "bioimpedance vector analysis"[Text Word] OR "biva"[Text Word] OR "phase angle"[Text Word] OR "extracellular water"[Text Word] OR "electrical impedance"[Text Word] OR "Electric Impedance"[MeSH Terms] OR ("blood volume monitor*"[Text Word] OR "plasma volume monitor*"[Text Word] OR "BVM"[Text Word] OR "biofeedback"[Text Word] OR "blood volume"[Text Word] OR "plasma volume"[Text Word] OR "crit line"[Text Word] OR "hematocrit"[Text Word]) OR ("lung ultraso*"[Text Word] OR "pleural ultraso*"[Text Word] OR "comet tail"[Text Word] OR "B line"[Text Word] OR "extravascular lung water"[Text Word] OR "LUS"[Text Word] OR "B-line"[Text Word] OR "B-lines"[Text Word] OR "B lines"[Text Word] OR "pulmonary echo*"[Text Word] OR "lung echo*"[Text Word])<br>**AND**<br>*Study design of interest*<br>(("randomized controlled trial"[Publication Type] OR "controlled clinical trial" [Publication Type] OR "randomized"[Tile/Abstract] OR "placebo"[Title/Abstract] OR "drug therapy"[MeSH Subheading] OR "randomly"[Title/Abstract] OR "trial"[Title/Abstract] OR "groups"[Title/Abstract] NOT ("animals"[MeSH Terms] NOT "humans"[MeSH	

Terms]))	
Scopus	
Population of interest<br>(((TITLE-ABS-KEY ("dialysis") OR TITLE-ABS-KEY ("hemodialysis") OR TITLE-ABS-KEY ("haemodialysis") OR TITLE-ABS-KEY ("peritoneal dialysis") OR TITLE-ABS-KEY ("renal replacement therapy") OR TITLE-ABS-KEY ("end stage renal failure") OR TITLE-ABS-KEY ("esrf") OR TITLE-ABS-KEY ("end stage renal disease") OR TITLE-ABS-KEY ("esrd") OR TITLE-ABS-KEY ("kidney failure") OR TITLE-ABS-KEY ("renal insufficiency") OR TITLE-ABS-KEY ("kidney insufficiency")))<br>**AND**<br>*Intervention of interest*<br>((TITLE-ABS-KEY ("volume assessment") OR TITLE-ABS-KEY ("volume management") OR TITLE-ABS-KEY ("volume control") OR TITLE-ABS-KEY ("volume overload") OR TITLE-ABS-KEY ("fluid overload") OR TITLE-ABS-KEY ("lung congestion") OR TITLE-ABS-KEY ("hypervolemia") OR TITLE-ABS-KEY ("overhydration") OR TITLE-ABS-KEY ("fluid status") OR TITLE-ABS-KEY ("weight adjustments") OR TITLE-ABS-KEY ("hydration status") OR TITLE-ABS-KEY ("target weight") OR TITLE-ABS-KEY ("dry weight") OR TITLE-ABS-KEY ("goal weight") OR TITLE-ABS-KEY ("ideal weight"))) AND (((TITLE-ABS-KEY ("bioimpedance") OR TITLE-ABS-KEY ("bio-impedance") OR TITLE-ABS-KEY ("bioimpedance analysis") OR TITLE-ABS-KEY ("bio-impedance analysis") OR TITLE-ABS-KEY ("bia") OR TITLE-ABS-KEY ("bioimpedance vector analysis") OR TITLE-ABS-KEY ("biva") OR TITLE-ABS-KEY ("phase angle") OR TITLE-ABS-KEY ("extracellular water") OR TITLE-ABS-KEY ("electrical impedance"))) OR ((TITLE-ABS-KEY ("blood volume monitor*") OR TITLE-ABS-KEY ("plasma volume monitor*") OR TITLE-ABS-KEY ("BVM") OR TITLE-ABS-KEY ("biofeedback") OR TITLE-ABS-KEY ("blood volume") OR TITLE-ABS-KEY ("plasma volume") OR TITLE-ABS-KEY ("crit line") OR TITLE-ABS-KEY ("hematocrit"))) OR ((TITLE-ABS-KEY ("lung ultraso*") OR TITLE-ABS-KEY ("pleural ultraso*") OR TITLE-ABS-KEY ("comet tail") OR TITLE-ABS-KEY ("B line") OR TITLE-ABS-KEY ("extravascular lung water") OR TITLE-ABS-KEY ("LUS") OR TITLE-ABS-KEY ("B-line") OR TITLE-ABS-KEY ("B-lines") OR TITLE-ABS-KEY ("B lines") OR TITLE-ABS-KEY ("pulmonary echo*") OR TITLE-ABS-KEY ("lung echo*")))))<br>**AND**<br>*Study design of interest*<br>((TITLE-ABS-KEY ("randomized controlled trial") OR TITLE-ABS-KEY ("controlled clinical trial") OR TITLE-ABS-KEY ("randomized") OR TITLE-ABS-KEY ("placebo") OR TITLE-ABS-KEY ("randomly") OR TITLE-ABS-	**718**

KEY ("trial") OR TITLE-ABS-KEY ("groups") OR TITLE-ABS-KEY ("drug therapy") AND NOT TITLE-ABS-KEY ("animal")))	
CENTRAL	**310**
Population of interest<br>"dialysis" OR "hemodialysis" OR "haemodialysis" OR "peritoneal dialysis" OR "renal replacement therapy" OR "end stage renal failure" OR "esrf" OR "end stage renal disease" OR "esrd" OR "kidney failure" OR "renal insufficiency" OR "kidney insufficiency"<br>AND<br>*Intervention of interest*<br>"volume assessment" OR "volume management" OR "volume control" OR "volume overload" OR "fluid overload" OR "lung congestion" OR "hypervolemia" OR "overhydration" OR "fluid status" OR "weight adjustments" OR "hydration status" OR "target weight" OR "dry weight" OR "goal weight" OR "ideal weight" AND "bioimpedance" OR "bio-impedance" OR "bioimpedance analysis" OR "bio-impedance analysis" OR "bia" OR "bioimpedance vector analysis" OR "biva" OR "phase angle" OR "extracellular water" OR "electrical impedance" OR "blood volume monitor$" OR "plasma volume monitor$" OR "BVM" OR "biofeedback" OR "blood volume" OR "plasma volume" OR "crit line" OR "hematocrit" OR "lung ultraso$" OR "pleural ultraso$" OR "comet tail" OR "B line" OR "extravascular lung water" OR "LUS" OR ("B-line") OR "B-lines" OR "B lines" OR "pulmonary echo$" OR "lung echo$"<br>*Study design of interest*<br>-	

Table S2. GRADE evidence profile for the efficacy and safety of technology-guided dry weight adjustment among dialysis patients.

Certainty assessment							Certainty	Importance
**№ of studies**	**Study design**	**Risk of bias**	**Inconsistency**	**Indirectness**	**Imprecision**	**Other considerations**		
**All-cause mortality**								
13	RCTs	serious	not serious	not serious	not serious	none	⊕⊕⊕□ Moderate	CRITICAL
**Cardiovascular events**								
9	RCTs	serious	not serious	not serious	serious	none	⊕⊕□□ Low	CRITICAL
**Hospitalization**								
5	RCTs	very serious	not serious	not serious	serious	none	⊕□□□ Very low	IMPORTANT
**Systolic blood pressure**								
13	RCTs	serious	serious	not serious	serious	none	⊕□□□ Very low	IMPORTANT
**Diastolic blood pressure**								
12	RCTs	serious	serious	not serious	serious	none	⊕□□□ Very low	IMPORTANT
**Left ventricular mass indexed for body surface area**								
6	RCTs	serious	not serious	not serious	serious	none	⊕⊕□□ Low	IMPORTANT
**Left ventricular mass indexed for height**								
2	RCTs	serious	not serious	not serious	serious	none	⊕⊕□□ Low	IMPORTANT
**Left ventricular ejection fraction**								
6	RCTs	serious	not serious	not serious	serious	none	⊕⊕□□ Low	IMPORTANT
**Pulse wave velocity**								
6	RCTs	serious	serious	not serious	not serious	none	⊕⊕□□ Low	IMPORTANT
**Intradialytic hypotension**								

Certainty assessment							Certainty	Importance
**№ of studies**	**Study design**	**Risk of bias**	**Inconsistency**	**Indirectness**	**Imprecision**	**Other considerations**		
5	RCTs	serious	serious	not serious	serious	none	⨁◯◯◯ Very low	CRITICAL
**Muscle cramp**								
5	RCTs	serious	not serious	not serious	serious	none	⨁⨁◯◯ Low	NOT IMPORTANT
**Cardiac arrhythmia**								
3	RCTs	serious	not serious	not serious	serious	none	⨁⨁◯◯ Low	IMPORTANT
**Vascular access problems**								
4	RCTs	serious	not serious	not serious	serious	none	⨁⨁◯◯ Low	IMPORTANT

Abbreviations; GRADE, Grading of Recommended Assessment, Development, and Evaluation approach; RCTs, randomized controlled trials.

Table S3. Sub-group analyses examining the effect of technology-guided dry weight adjustment on cardiovascular events

**Subgroup analyses**	No. of studies	No. of patients	Pooled risk ratio (95% CI)	P-values	Assessment of heterogeneity	
					I^2 index	P-value
Dialysis modalities						
PD	4	643	0.83 (0.54-1.29)	0.42	0%	0.60
HD	5	1512	0.78 (0.66-0.92)	<0.01	7.6%	0.36
Race						
Asian	7	1727	0.76 (0.57-1.03)	0.07	2.7%	0.41
Non-Asian	2	428	0.79 (0.71-0.89)	<0.01	0%	0.89
Tools of dry weight assessment						
BIA	8	1792	0.76 (0.58-1.01)	0.06	0%	0.52
LUS	1	363	0.80 (0.71-0.89)	<0.01	0%	1.00
Study follow-up time						
≤1 years	5	896	0.68 (0.46-0.98)	0.04	0%	0.47
>1 years	4	1259	0.80 (0.72-0.89)	<0.01	0%	0.58
Dialysis vintage						
Incident	1	445	0.44 (0.14-1.45)	0.18	0%	1.00
Prevalent	8	1710	0.79 (0.72-0.88)	<0.01	0%	0.62
Risk of bias						
High	3	500	0.82 (0.47-1.43)	0.48	0%	0.42
Low	2	711	0.77 (0.57-1.04)	0.09	6.2%	0.30
Some concerns	4	944	0.77 (0.54-1.10)	0.16	11.6%	0.33

Abbreviation; BIA; bioimpedance analysis; HD, hemodialysis; LUS, lung ultrasound; PD, peritoneal dialysis.

Table S4. Sub-group analyses examining the effect of technology-guided dry weight adjustment on intradialytic hypotension

**Subgroup analyses**	No. of studies	No. of patients	Rate ratios (95% CI)	P-values	Assessment of heterogeneity	
					I^2 index	P-value
Race						
Asian	2	348	0.92 (0.77-1.10)	0.37	45.7%	0.17
Non-Asian	3	738	0.94 (0.77-1.16)	0.58	74.8%	<0.01
Tools of dry weight assessment						
BIA	3	473	0.96 (0.89-1.04)	0.30	0%	0.37
LUS	1	363	0.83 (0.76-0.91)	<0.01	0%	1.00
BIA + LUS	1	250	1.04 (0.96-1.14)	0.33	0%	1.00
Study follow-up time						
≤1 years	3	473	0.96 (0.89-1.04)	0.30	0%	0.37
>1 years	2	613	0.93 (0.75-1.16)	0.53	92.6%	<0.01
Risk of bias						
Low	1	363	0.83 (0.76-0.91)	<0.01	0%	1.00
Some concerns	4	723	0.99 (0.92-1.07)	0.83	24.4%	0.27

Abbreviation; BIA; bioimpedance analysis; BVM; blood volume monitoring; LUS, lung ultrasound.

Table S5. Sub-group analyses examining the effect of technology-guided dry weight adjustment on systolic blood pressure

**Subgroup analyses**	No. of studies	No. of patients	WMD (95% CI)	P-values	Assessment of heterogeneity	
					I^2 index	P-value
Dialysis modalities						
PD	6	1,081	-2.23 (-5.33, 0.86)	0.16	34.7%	0.15
HD	7	748	-2.02 (-6.00, 1.96)	0.32	78.5%	<0.01
Race						
Asian	6	914	-3.16 (-5.51, -0.82)	0.01	0%	0.55
Non-Asian	6	625	-0.34 (-4.93, 4.25)	0.88	78.2%	<0.01
Mixed	1	290	-2.84 (-7.73, 2.04)	0.25	0%	1.00
Tools of dry weight assessment						
BVM	1	160	-6.08 (-12.6, 0.44)	0.07	0%	1.00
BIA	11	1,598	-1.60 (-4.48, 1.27)	0.27	73.8%	<0.01
LUS	1	71	-2.91 (-12.32, 6.50)	0.54	0%	1.00
Study follow-up time						
≤1 years	10	1,348	-3.05 (-5.45, -0.64)	0.01	22.4%	0.22
>1 years	3	481	-0.05 (-4.68, 4.59)	0.98	84.1%	<0.01
Risk of bias						
High	4	549	-1.66 (-8.12, 4.79)	0.61	72.1%	0.01
Low	4	629	-2.87 (-5.74, -0.01)	0.05	0%	0.68
Some concerns	5	651	-1.04 (-5.19, 3.11)	0.62	76.8%	<0.01

Abbreviation; BIA; bioimpedance analysis; BVM; blood volume monitoring; HD, hemodialysis; LUS, lung ultrasound; PD, peritoneal dialysis.

Table S6. Sub-group analyses examining the effect of technology-guided dry weight adjustment on diastolic blood pressure

**Subgroup analyses**	No. of studies	No. of patients	WMD (95% CI)	P-values	Assessment of heterogeneity	
					I^2 index	P-value
Dialysis modalities						
PD	6	1,081	-0.23 (-2.77, 2.32)	0.86	63.6%	0.01
HD	6	617	-0.85 (-2.85, 1.16)	0.41	65.1%	0.01
Race						
Asian	6	914	-1.83 (-3.34, -0.32)	0.02	20.1%	0.28
Non-Asian	5	494	1.20 (-1.81, 4.22)	0.43	66.9%	0.02
Mixed	1	290	-0.04 (-4.59, 4.51)	0.99	58.1%	0.09
Tools of dry weight assessment						
BVM	1	160	-3.22 (-7.30, 0.86)	0.12	0%	1.00
BIA	10	1,467	-0.31 (-1.87, 1.24)	0.69	64.4%	<0.01
LUS	1	71	-3.40 (-9.97, 3.17)	0.31	0%	1.00
Study follow-up time						
≤1 years	9	1,217	-0.87 (-2.71, 0.97)	0.35	57.4%	0.01
>1 years	2	481	0.77 (0.14, 1.40)	0.02	0%	0.65
Risk of bias						
High	4	549	0.38 (-3.59, 4.35)	0.85	73.2%	0.01
Low	3	498	-1.72 (-4.92, 1.47)	0.29	50.2%	0.09
Some concerns	5	651	-0.29 (-2.20, 1.62)	0.77	63.5%	0.03

Abbreviation; BIA; bioimpedance analysis; BVM; blood volume monitoring; HD, hemodialysis; LUS, lung ultrasound; PD, peritoneal dialysis.

Table S7 Sub-group analyses examining the effect of technology-guided dry weight adjustment on pulse wave velocity

**Subgroup analyses**	No. of studies	No. of patients	WMD (95% CI)	P-values	Assessment of heterogeneity	
					I^2 index	P-value
Dialysis modalities						
PD	2	184	-5.51 (-14.09, 3.79)	0.26	99.7%	<0.01
HD	4	663	-1.03 (-2.28, 0.21)	0.10	93.6%	<0.01
Race						
Asian	2	293	-4.95 (-14.28, 4.38)	0.30	99.6%	<0.01
Non-Asian	4	554	-1.12 (-2.28, 0.04)	0.06	94.1%	<0.01
Tools of dry weight assessment						
BIA	5	606	-2.99 (-5.97, -0.01)	0.05	98.8%	<0.01
BIA + LUS	1	241	0.30 (0.03, 0.57)	0.03	0%	1.00
Study follow-up time						
≤1 years	4	475	-3.19 (-6.89, 0.51)	0.09	99.1%	<0.01
>1 years	2	372	0.91 (-3.36, 1.54)	0.47	96.2%	<0.01
Risk of bias						
High	2	182	-1.39 (-3.05, 0.27)	0.10	88.7%	<0.01
Low	2	268	-5.96 (-13.33, 1.41)	0.11	99.2%	<0.01
Some concerns	2	397	0.13 (-0.33, 0.60)	0.57	51.9%	0.15

Abbreviation; BIA; bioimpedance analysis; HD, hemodialysis; LUS, lung ultrasound; PD, peritoneal dialysis.
